# RNF4 interacts with multiSUMOylated ETV4

**DOI:** 10.12688/wellcomeopenres.9935.2

**Published:** 2017-02-17

**Authors:** Elisa Aguilar-Martinez, Baoqiang Guo, Andrew D. Sharrocks

**Affiliations:** 1 Faculty of Biology, Medicine and Health, University of Manchester, Manchester, M13 9PT, UK

**Keywords:** SUMO, SIM, RNF4, ETV4

## Abstract

Protein SUMOylation represents an important regulatory event that changes the activities of numerous proteins. Recent evidence demonstrates that polySUMO chains can act as a trigger to direct the ubiquitin ligase RNF4 to substrates to cause their turnover through the ubiquitin pathway. RNF4 uses multiple SUMO interaction motifs (SIMs) to bind to these chains. However, in addition to polySUMO chains, a multimeric binding surface created by the simultaneous SUMOylation of multiple residues on a protein or complex could also provide a platform for the recruitment of multi-SIM proteins like RNF4. Here we demonstrate that multiSUMOylated ETV4 can bind to RNF4 and that a unique combination of SIMs is required for RNF4 to interact with this multiSUMOylated platform. Thus RNF4 can bind to proteins that are either polySUMOylated through a single site or multiSUMOylated on several sites and raises the possibility that such multiSIM-multiSUMO interactions might be more widespread.

## Introduction

SUMOylation is a process whereby proteins are modified by SUMO (small ubiquitin-like modifier) through conjugation to specific lysine residues. This type of posttranslational modification has been increasingly recognised as an important regulatory event in many cellular processes and is often deregulated in disease situations (reviewed in Yang & Chiang, 2013). Much of the focus has been on the role of SUMO in regulating nuclear processes such as DNA repair, chromatin structural changes and transcriptional regulation (reviewed in Cubeñas-Potts & Matunis, 2013; Jackson & Durocher, 2013). The addition of SUMO to a protein constitutes a large change in overall protein size and as such, one major mode of action is through providing an additional protein binding surface which changes the repertoire of potential interacting proteins (reviewed in Hay, 2013). Binding partners are attracted to SUMO through short hydrophobic regions known as SIMs (SUMO interacting motifs) ( Hecker *et al.*, 2006; Song *et al.*, 2004). Proteins are typically modified by a single SUMO molecule by conjugation to specific lysine residues. However while this is the case for SUMO1, both SUMO2 and SUMO3 can develop SUMO chains through conjugation of additional SUMO moieties through a lysine residue located within the N-terminal region of these proteins (reviewed in Ulrich, 2008). This leads to the deposition of SUMO chains on substrates which provide a polymeric binding surface for targeting new protein-protein interactions. This type of interaction has been exploited by proteins like RNF4 which has multiple SIMs which can simultaneously interact with several SUMO moieties in polymeric SUMO chains ( Keusekotten *et al.*, 2014; Kung *et al.*, 2014; Tatham *et al.*, 2008; Xu *et al.*, 2014).

RNF4 is a SUMO targeted ubiquitin ligase (STUbL) which is recruited by polySUMOylated substrates like PML and subsequently targets PML for degradation ( Lallemand-Breitenbach *et al.*, 2008; Tatham *et al.*, 2008; reviewed in Gärtner & Muller, 2014; Praefcke *et al.*, 2012). Since this initial discovery, RNF4 has been shown to play an essential role in DNA repair ( Galanty *et al.*, 2012; Yin *et al.*, 2012) and further RNF4 substrates have been identified such as FANCI and FANCD2 which function in the DNA repair pathways ( Gibbs-Seymour *et al.*, 2015). The role of RNF4 has been broadened by the identification of substrates involved in chromatin and transcription regulation such as TRIM28/KAP1 ( Kuo *et al.*, 2014) and ETV4 ( Guo & Sharrocks, 2009). In these contexts, it is assumed that RNF4 elicits its functions through polySUMO chains. However, it is possible that other multivalent interaction modalities might be employed. Indeed, rather than one modification of individual component, efficient DNA repair requires SUMOylation of many different proteins involved in homologous recombination ( Psakhye & Jentsch, 2012). It is generally assumed that polySUMOylation is the driving factor but this finding raises the possibility that multiSUMOylation of many components of a single protein complex might instead present a binding surface for recruiting RNF4. Alternatively, RNF4 might be recruited to substrates by binding to a multi-SUMO platform created by the simultaneous SUMOylation of multiple lysine residues in the same proteins. Indeed many proteins have the potential to be multiSUMOylated due to having multiple consensus sites for SUMOylation ( Yang *et al.*, 2006), and proteome-wide studies have shown that large numbers of proteins contain two or more SUMO2 modified lysine residues ( Hendriks *et al.*, 2014; Tammsalu *et al.*, 2014). Conversely, other proteins could potentially recognise multi- or polySUMO platforms through utilising multiple SIMs in an analogous manner to RNF4. Recently, through the use of an artificial multi-SUMO scaffold we identified dozens of multiSUMO binding proteins, one of which, the transcriptional regulator ZMYM2, was investigated in detail and shown to use multiple SIMs to interact with this scaffold ( Aguilar-Martinez *et al.*, 2015).

To further our understanding of multimeric SUMO interactions, we investigated the requirements for RNF4 binding to SUMOylated ETV4 (otherwise known as PEA3 and E1AF). ETV4 is an ETS transcription factor and represents an example of a protein with multiple SUMO modification sites which forms high molecular weight SUMOylated species ( Guo & Sharrocks, 2009; Nishida *et al.*, 2007). SUMOylation was previously shown to be important for ETV4 transactivation activity but also triggers its degradation in a temporally delayed manner following growth factor-mediated signalling through the ERK pathway ( Guo & Sharrocks, 2009). The STUbL RNF4 was shown to play an important role in controlling both ETV4-mediated target gene activation and ETV4 degradation through the ubiquitin pathway. Here, we investigated whether multiSUMOylation could provide a means for driving RNF4 recruitment to ETV4. Using a series of binding assays we show that multiSUMOylation can promote the binding of the multi-SIM containing protein RNF4 to ETV4.

## Materials and methods

### Plasmid constructs

The following plasmids were used in mammalian cell transfections; pSG5-PIAS4 (encoding Myc-tagged PIAS4)(kindly provided by Frances Fuller-Pace; Jacobs *et al.*, 2007), pCDNA3-Ubc9/UBE2I, pCDNA3-His-SUMO-3 and pCDNA3-His-SUMO-3(K11R) (kindly provided by Ron Hay; Tatham *et al.*, 2001). pAS981 (encodes full-length flag-tagged zebrafish ETV4/PEA3 cloned in the XbaI/KpnI sites of pCDNA3; constructed by Amanda Greenall).

The following plasmids were used for bacterial expression. MBP-RNF4(WT), MBP-RNF4(SIM1mut), MBP-RNF4(SIM2mut), MBP-RNF4(SIM3mut), MBP-RNF4(SIM4mut), MBP-RNF4(SIM1,2mut), MBP-RNF4(SIM1,2,3mut), and MBP-RNF4(SIM1,2,3,4mut) all in pLou3 (kindly provided by Ron Hay; Tatham *et al.*, 2008) have been described previously. MBP-RNF4(SIM1,4mut) (pAS2760) and MBP-RNF4(SIM2,3mut) (pAS2761) were constructed by Quikchange mutagenesis (Stratagene), using the templates pLou-MBP-RNF4(SIM1mut) (pAS2753) and pLou-MBP-RNF4(SIM2mut) (pAS2754) and the primer pair combinations ADS2587/ADS2588 and ADS2583/ADS2584 respectively. pAS2502 [encoding GST-ETV4(1-335)(WT)] ( Guo *et al.*, 2011), has been described elsewhere. His-SUMO3-K11R (pAS2767) was constructed by inserting a NcoI-EcoRI-cleaved PCR fragment (generated using primer pair ADS2582/ADS2581 and the template pCDNA3-HA-SUMO2K11R; Tatham *et al.*, 2001) into the same sites in pET30b. pAS2501 [encoding GST-ETV4(1-480)(WT)] was constructed by Niki Panagiotaki by ligating a BamHI/EcoRI-cleaved PCR product (primers ADS1580/ADS1584 and template pAS1801) into the same sites in pGEXKG. pAS1801 contains the full-length mouse cDNA cloned into the HindIII and SalI sites of pCDNA3. pAS4159 [encoding GST-ETV4(1-480)(K12R)], pAS4160 [encoding GST-ETV4(1-480)(K34R)], pAS4161 [encoding GST-ETV4(1-480)(K1234R)] were constructed ligating BamHI/EcoRI-cleaved PCR products (primers ADS1580/ADS1584 and templates pAS1034, pAS1040 and pAS1037; Guo & Sharrocks, 2009) into the same sites in pGEXKG. Details of PCR primers are given in Table 1.

**Table 1.** PCR primers used in this study.

Primer	Sequence (5’-3’)
ADS1580	ATCGGGATCCATGGAGCGGAGGATGAAAGGC
ADS1584	ATCGGAATTCAGTAAGAATATCCACCTCTGTG
ADS2581	GCGGGGAATTCGGTAGTGGTAGTGGTAGTATGTCCGAGGAGAAGCCCAAG
ADS2582	GCGGGCCATGGCTATGTCCGAGGAGAAGCCCAAG
ADS2583	GTGAATCTTTAGAGCCTGCGGCTGCGGACCTGACTCACAATGA
ADS2584	TCATTGTGAGTCAGGTCCGCAGCCGCAGGCTCTAAAGATTCAC
ADS2587	GACTCACAATGACTCTGCTGCGGCTGCTGAAGAAAGGAGAAGGC
ADS2588	GCCTTCTCCTTTCTTCAGCAGCCGCAGCAGAGTCATTGTGAGTC

### Cell culture, co-immunoprecipitation analysis and western blotting

HEK293T cells were grown in DMEM supplemented with 10% foetal bovine serum and where indicated, cells were treated with phorbol 12-myristate (PMA)(Sigma)(10 nM) for 6 hours. Plasmid transfection was performed using Polyfect (Qiagen). Western blotting and immunoprecipitation were carried out with the primary antibodies; rabbit polyclonal anti-RNF4 (1:5,000 gift from Jorma Palvino; Häkli *et al.*, 2005), mouse monoclonal anti-Flag M2 (1:2,000 Sigma; F3165), and mouse monoclonal anti-MBP (1:1,000 Abcam, ab49923 { Figure 2A} and 1:1,000 Cell Signaling mouse monoclonal cat no 2396 { Figure 2B}). The proteins were detected as described previously ( Aguilar-Martinez *et al.*, 2015).

### Protein purification and GST pulldown assays

Recombinant proteins were expressed in *Escherichia coli* BL21 or BL21-CodonPlus(DE3)-RIL (Stratagene). To prepare SUMOylated recombinant GST-ETV4, a reconstituted SUMOylation system in *E. coli* was used ( Mencía & de Lorenzo, 2004). Recombinant GST-fusion proteins were purified and GST pulldown assays were carried out as described previously ( Aguilar-Martinez *et al.*, 2015).

## Results

**Figure 1.**
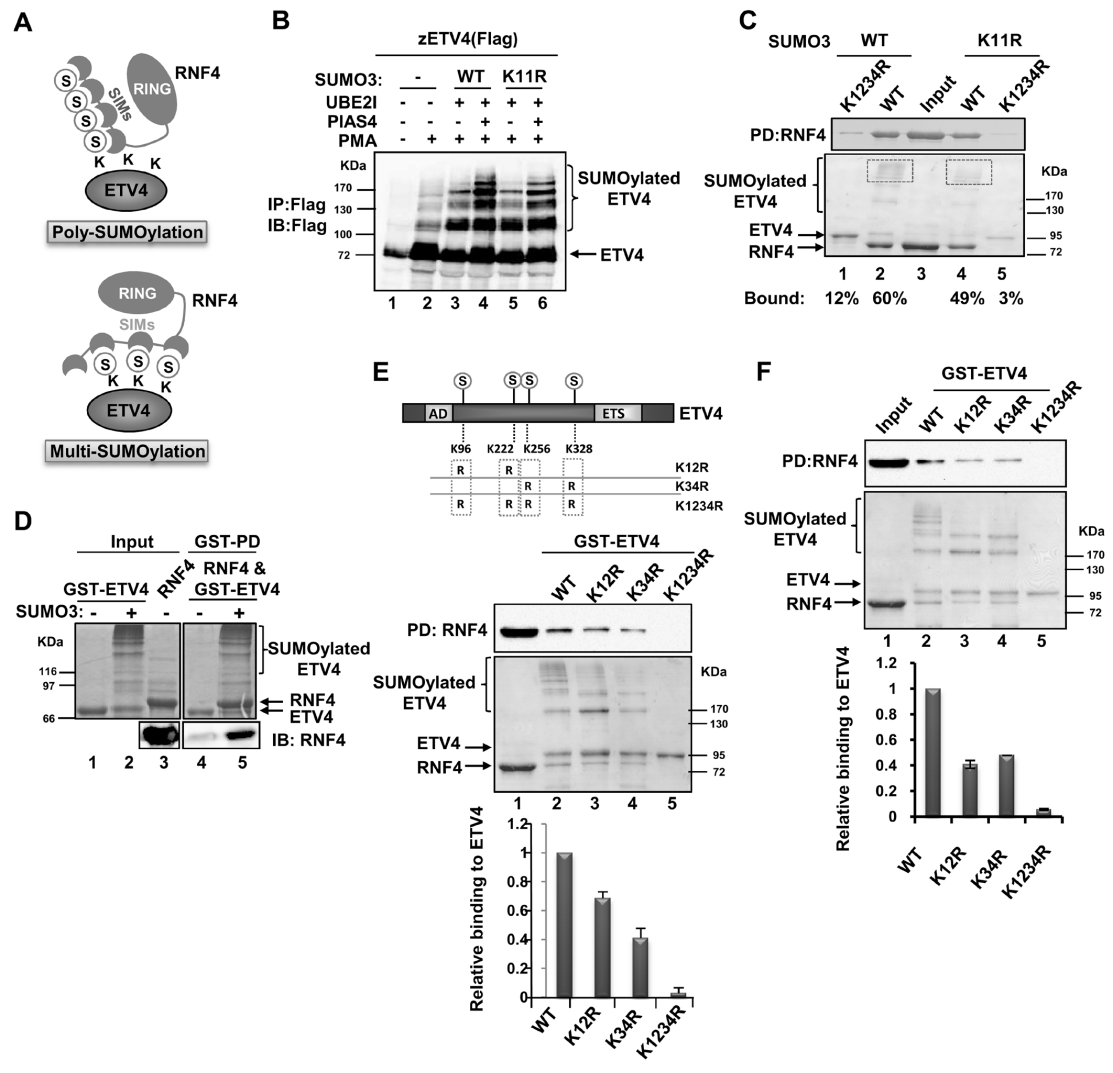
RNF4 binds to multiSUMOylated ETV4. ( **A**) Schematic representation of RNF4 interacting with a poly- or multiSUMOylated form of ETV4 through its SIMs. ( **B**) Flag-tagged zETV4 was immunoprecipitated (IP) with anti-Flag antibody from HEK293T cells co-transfected with wild-type (WT) SUMO3 or SUMO3(K11R), UBC9/UBE2I and PIAS4 where indicated. ETV4 was detected by immunoblotting (IB) with anti-Flag antibody. Where indicated, cells were treated with PMA for 6 h. ( **C**) GSTpulldown analysis of the interaction of recombinant MBP-RNF4 with GST-ETV4(full-length; WT or K1234R mutant) modified with SUMO3(WT) or SUMO3(K11R). Pulled down RNF4 was detected by immunoblotting with anti-RNF4 antibody (top panel; pulldown [PD]). Bottom panel: Ponceau stained nitrocellulose membrane. Boxes highlight the presence or absence of high molecular weight SUMO chains. Percentages of binding indicate the binding of RNF4 relative to input. ( **D**) GST pulldown (GST-PD) analysis of MBP-RNF4 binding to non-SUMOylated or SUMOylated recombinant GST-ETV4(1-355). Top: Coomassie stained gel of the input proteins (lanes 1–3) and results of the pulldown (lanes 4–5). Bottom: immunoblot of the pulldown (PD) using anti-RNF4 antibody. ( **E** and **F**) GST pulldown (PD) analysis of the interaction of recombinant MBP-RNF4 with the indicated WT and mutant forms of GST-ETV4(full-length) modified with SUMO3(WT)( **E**) or SUMO3(K11R)( **F**). Top: schematic of ETV4 showing its SUMOylation sites and locations of the amino acid substitutions in the ETV4 mutants. Middle: GST pulldown assay with RNF4 binding shown in the top panel using an anti-RNF4 antibody (pulldown [PD]) and a Ponceau stained membrane at the bottom showing the GST bait and input proteins. Bottom: Quantification of RNF4 binding to the different SUMOylated (WT) forms of ETV4 (relative to input, taken as 1). The SUMOylated GST-ETV4 species in ( **C**– **F**) were generated using an *in vivo* recombinant protein bacterial expression system ( Mencia & Lorenzo, 2004). Molecular weight markers are shown on coomassie and Ponceau stained gels.

ETV4 contains five evolutionarily conserved sites which fit the core SUMO consensus sequence ψKxE. We previously showed that the three most N-terminally located sites in mouse ETV4 (K96, K222 and K256) are the major sites for modification with SUMO ( Guo & Sharrocks, 2009). It is assumed that RNF4 binds to proteins containing polySUMO chains, through its multiple SIM motifs. However, it is equally plausible that RNF4 might be able to recognise multiple single SUMO moieties conjugated to different sites in ETV4 ( Figure 1A). Indeed, the latter possibility is supported by the observation that RNF4-mediated polyubiquitination *in vivo* is diminished as the number of SUMO conjugation sites in ETV4 are reduced ( Guo & Sharrocks, 2009).

**Figure 2.**
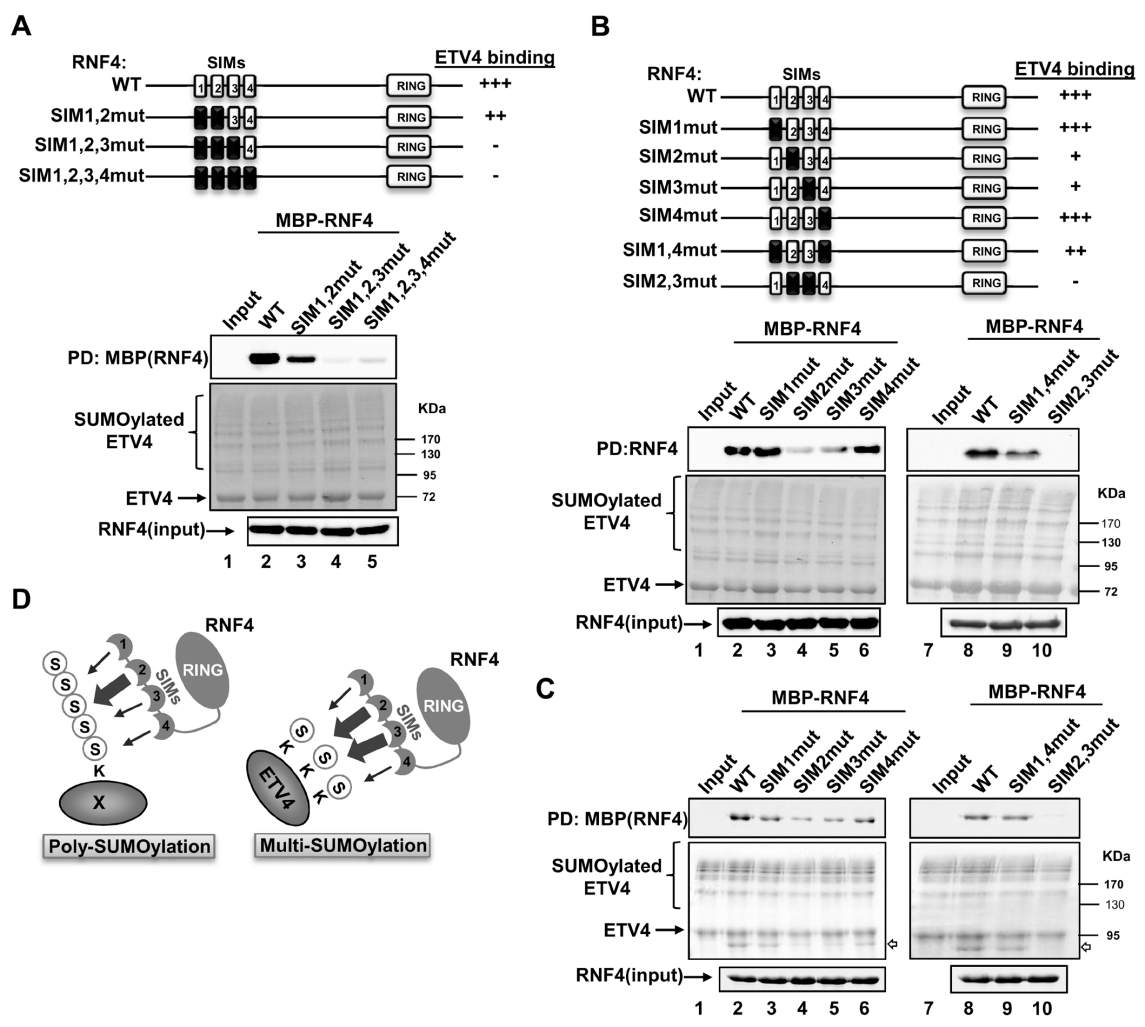
Mapping the SIM motifs in RNF4 for binding to multiSUMOylated ETV4. ( **A**– **C**) GST pulldown assays of the indicated wild-type and mutant forms of MBP-RNF4 binding to ETV4(1-355) SUMOylated with wild-type SUMO3 ( **A** and **B**) or full length ETV4 with SUMO3(K11R)( **C**). Schematic representations of wild type (WT) and mutant forms of RNF4 are shown at the top. Symbols on the right show the binding of the different RNF4 forms relative to RNF4(WT) (-, ≤10%), (+, <40%), (++, ≥50%) and (+++, ≥80%). RNF4 binding (top panels; pulldown [PD]) and input proteins (bottom panels) were detected by immunoblotting using an anti-MBP antibody. SUMOylated GST-ETV4 is shown by Ponceau staining (middle panels). Open arrows in ( **C**) indicate the band corresponding to MBP-RNF4. Molecular weight markers are shown on Ponceau stained gels. ( **D**) Schematic representation of RNF4 interacting with a polySUMOylated protein (X) or multiSUMOylated form of ETV4 through its SIMs. Arrows represent SIM-SUMO interactions, with larger arrows denoting the dominant SIM-SUMO interactions.

We first examined whether we could find evidence for multi- or polySUMOylation of ETV4 *in vivo*. We examined ETV4 SUMOylation status following co-transfection of cells with UBE2I/UBC9 and PIAS4 (to maximise SUMOylation levels) and either wild-type SUMO3 or the SUMO3(K11R) mutant. This latter form of SUMO cannot form polySUMO chains ( Tatham *et al.*, 2001). Cells were treated with PMA to enhance the levels of SUMOylation ( Guo *et al.*, 2009). Multiple high molecular weight SUMO conjugates were observed in the presence of wild-type SUMO3 ( Figure 1B, lanes 3 and 4) and an identical pattern of conjugation was observed in the presence of SUMO3(K11R) ( Figure 1B, lanes 5 and 6). The multiple bands arising are likely due to a combination of single site SUMOylation events and multi-site SUMOylation to produce the higher molecular weight lower mobility species. Thus, as SUMO3(K11R) gives the same banding pattern as wild-type SUMO3, the multiple species that we observe in cells likely represent different combinations of multiSUMOylated ETV4 rather than polySUMOylated conjugates.

Next we explored whether multiSUMOylation is sufficient for binding of RNF4 to ETV4. To produce SUMOylated protein, full-length ETV4 was co-expressed in bacteria as a GST fusion protein along with the SUMO conjugation machinery and either wild-type SUMO3 or SUMO3(K11R) and in both cases, multiple SUMOylated species were observed, with wild-type SUMO3 generating substantially more of the higher molecular weight conjugates, indicative of polySUMOylation ( Figure 1C, lower panel). Note that the banding pattern was not identical to Flag-tagged ETV4 in mammalian cells (Figure 1B), likely due to the differing mobilities of the branched chain SUMO conjugates associated with differentially tagged ETV4 constructs (N-terminal large GST tag versus a short C-terminal Flag tag). We then tested the binding of recombinant RNF4 to these SUMOylated forms of GST-ETV4 and found that both forms of SUMOylated ETV4 were able to efficiently bind to recombinant RNF4 in GST pulldown assays ( Figure 1C, lanes 2 and 4; top panel) indicating that polySUMOylation is not required for efficient RNF4 binding. Although the levels of multi-SUMOylation are low, this is sufficient for RNF4 binding as mutant forms of ETV4 that are defective in SUMOylation cannot recruit RNF4 ( Figure 1C, lanes 1 and 5). Importantly, we further verified that ETV4 SUMOylation is required for efficient RNF4 binding ( Figure 1D) ruling out SUMO-independent binding of RNF4 to ETV4. To further extend these findings, we removed pairs of SUMOylation sites in ETV4 to reduce the potential for multi-SUMOylation. Loss of these SUMO modification sites reduced the levels of ETV4 multiSUMOylation, and provoked a concomitant reduction in RNF4 binding ( Figure 1E). A virtually identical effect was observed for binding to ETV4 mutants modified with SUMO3(K11R) further demonstrating that SUMO chain formation is not required for RNF4 binding ( Figure 1F). Although we have not determined the sites of SUMO modification on ETV4 in the bacterially generated SUMOylated ETV4, the loss of SUMOylation in the ETV4(K1234R) mutant that lacks all of the mapped SUMO conjugation sites ( Guo *et al.*, 2009) demonstrates that SUMOylation is taking place at the correct lysine residues rather than at cryptic sites ( Figure 1E and F, bottom panels). Thus RNF4 binding is diminished by reducing the potential for ETV4 multi-SUMOylation by either removing SUMO conjugation sites or using a chain forming mutant version of SUMO.

RNF4 has previously been shown to require multiple SIM motifs for binding to polySUMO chains. We therefore asked whether the multiple SIMs in RNF4 are required for binding to SUMO conjugated ETV4. We created multi-SUMOylated ETV4 by expressing GST-ETV4 along with the SUMO3 ( Figure 2A and B). We then tested the interaction of multi-SUMOylated ETV4 in GST pulldown assays with RNF4 proteins containing mutations in one or more of its SIMs in RNF4. Mutating different combinations of two or more SIMs reduced binding to multiSUMOylated ETV4 ( Figure 2A and B), with the simultaneous mutation of SIMs 2 and 3 eliminating detectable binding ( Figure 2B, lane 10). We then asked whether any of the individual SIMs are important for binding to multiSUMOylated ETV4 and found that mutation of either SIM2 or SIM3 substantially reduced RNF4-ETV4 interactions while minimal effects were seen after mutating SIMs 1 or 4 ( Figure 2B, lanes 3–6). These findings are broadly in keeping with the requirements determined for RNF4 binding to polySUMO chains, where SIM2 plays the most important role ( Tatham *et al.*, 2008). However, mutation of SIM3 alone in RNF4 had little impact on RNF4 binding to polySUMO chains ( Tatham *et al.*, 2008) indicating that SIM3 appears to play a more prominent role in binding to multiSUMOylated ETV4. Importantly, we got virtually identical results when wild-type SUMO3 was replaced with the mutant SUMO3(K11R) that is defective in polySUMO chain formation ( Figure 2C). Thus, RNF4 can make differential use of its SIMs to potentially bind to both multi- and polySUMOylated proteins ( Figure 2D).

Collectively, these findings on ETV4-RNF4 interactions establish that multiSUMOylation can act as a platform to recruit a multi-SIM domain containing protein.

Raw Data for Figures 1 & 2 **Figure 1 raw data. **The complete western blots are shown and the areas taken for inclusion in the panels in Figure 1 are highlighted (indicated by boxes).
**Figure 2A and C. Raw data.** The complete western blots are shown and the areas taken for inclusion in the panels in Figure 2 are highlighted (indicated by boxes).
**Figure 2B. Raw data.** The complete western blots are shown and the areas taken for inclusion in the panels in Fig. 2 are highlighted (indicated by boxes). Click here for additional data file. Copyright: © 2017 Aguilar-Martinez E et al. 2017 Data associated with the article are available under the terms of the Creative Commons Zero "No rights reserved" data waiver (CC0 1.0 Public domain dedication).

## Discussion

One of the major mechanisms through which SUMOylation affects target protein activity is through providing a binding platform for other proteins. This can be elicited through the conjugation of single SUMO moieties, or through the subsequent formation of polySUMO chains on top of these initial conjugation events. These polySUMO chains act as a binding platform for STUbLs such as RNF4, which subsequently target the SUMOylated protein for ubiquitination and degradation ( Boutell *et al.*, 2011; Lescasse *et al.*, 2013; Sun & Hunter, 2012; Tatham *et al.*, 2008).

However, in addition to presenting SUMO in the form of chains, multimeric SUMO platforms can be created through the simultaneous modification of several residues at the same time, either on a single protein or several proteins in a complex. We recently provided evidence for this possibility through the use of an artificial multiSUMO scaffold and identified dozens of proteins which are recruited by this scaffold ( Aguilar-Martinez *et al.*, 2015). Here we have approached the problem from the other direction and asked whether a multiSUMOylated substrate can act as a binding platform for RNF4. Using multiSUMOylated ETV4 as a binding platform, we demonstrate that RNF4 can be recruited in a multiSUMO-dependent manner. Thus RNF4 can potentially be recruited to multimeric SUMO scaffolds created through polySUMO chains, multiSUMO platforms ( Figure 2D), or even a combination of both. Indeed, the prospect of a combination of multi- and polySUMOylation involvement in STUbL targeting, is attractive as the availability of more SUMO modification sites increases the probability of chain formation.

 RNF4 contains multiple SIMs for binding to polySUMO chains ( Keusekotten *et al.*, 2014; Kung *et al.*, 2014; Tatham *et al.*, 2008). Of the four SIM motifs, SIM2 is the most important and is thought to nucleate the binding of the other SIMs to the polySUMO chains. This type of behaviour has led to the concept of one or a subset of SIMs being designated as “dominant” ( Sun & Hunter, 2012). More recently, a structural study confirmed an important role for SIM2 in polySUMO binding by RNF4 but also provided evidence for a supporting substantive role for SIM3 ( Xu *et al.*, 2014). Here we show that both SIM2 and SIM3 play dominant roles in the case of RNF4 binding to multiSUMOylated ETV4. This is unsurprising given the likely different stereospecific presentation of the multimeric SUMO binding platforms in the form of polySUMO chains and through the presentation of multiple SUMO moieties on the surface of ETV4. Thus for RNF4 the linear series of closely spaced SIMs might be optimally configured to interact with polySUMO chains and a select subset of multiSUMO platforms while other proteins might have different binding modes. Indeed, we demonstrated that the multiple SIMs in ZMYM2 are not linearly arranged in the protein but are spread throughout a large region ( Aguilar-Martinez *et al.*, 2015). Again one of these SIMs is “dominant” and presumably helps nucleate a multi-SIM scaffold that recognises a unique subset of multiSUMO substrates. Conversely from the substrate perspective, the presentation of the conjugated SUMO moieties following multiSUMOylation is unlikely to be ordered in a linear fashion as seen in polySUMO chains. Indeed, the SUMOylation sites in ETV4 are spread across a 330 amino acid region in its N-terminal region. This means that to provide a clustered array that can be simultaneously recognised by the tandem SIMs RNF4, the N-terminal region of ETV4 must exhibit considerable flexibility. The lack of recognisable ordered domains in this region would facilitate such flexibility.

SUMOylation of ETV4 triggers both its activation and subsequent degradation, and we have also shown that RNF4 is also important for both processes ( Guo & Sharrocks, 2009). Thus, multiSUMOylation could provide an important link in this sequence of events. In addition, multiSUMOylated ETV4 might also recruit other multi-SIM domain containing proteins during the activation process allowing the sequential binding of a coactivator followed by exchange for RNF4. This would provide an attractive mechanism for the coordinated activation and subsequent inactivation of ETV4. The requirement for mutiSUMOylation would effectively set a threshold which triggers progression along the activation-inactivation pathway and enable temporal control over ETV4-mediated transcriptional activation activity.

In summary, we have provided evidence that in addition to its documented polySUMO binding activities, RNF4 has the potential to bind to multiSUMOylated substrates such as ETV4. This has broader significance as it opens up the possibilities of more widespread regulatory interactions between multiSUMOylated proteins and multi-SIM domain containing protein partners.

## Data availability

The data referenced by this article are under copyright with the following copyright statement: Copyright: © 2017 Aguilar-Martinez E et al.

Data associated with the article are available under the terms of the Creative Commons Zero "No rights reserved" data waiver (CC0 1.0 Public domain dedication). 

*Figshare:* Raw data for Figures 1 and 2. doi: https://doi.org/10.6084/m9.figshare.4641067.v1 ( Aguilar-Martinez *et al.*, 2016).
