# Supplementary material for: RNF4 interacts with multiSUMOylated ETV4
Source: Wellcome Open Res. 2017 Feb 17;1:3. Originally published 2016 Nov 15. [Version 2] doi: 10.12688/wellcomeopenres.9935.2 (PMC5445624; doi:10.12688/wellcomeopenres.9935.2)
Supplement: Raw Data for Figures 1 & 2 — Figure 1 raw data. The complete western blots are shown and the areas taken for inclusion in the panels in Figure 1 are highlighted (indicated by boxes). Figure 2A and C. Raw data. The complete western blots are shown and the areas taken for inclusion in the panels in Figure 2 are highlighted (indicated by boxes). Figure 2B. Raw data. The complete western blots are shown and the areas taken for inclusion in the panels in Fig. 2 are highlighted (indicated by boxes). [file wellcomeopenres-1-11629-s0000.tgz › Raw_data_paper_revised.pptx]

## Slide 1
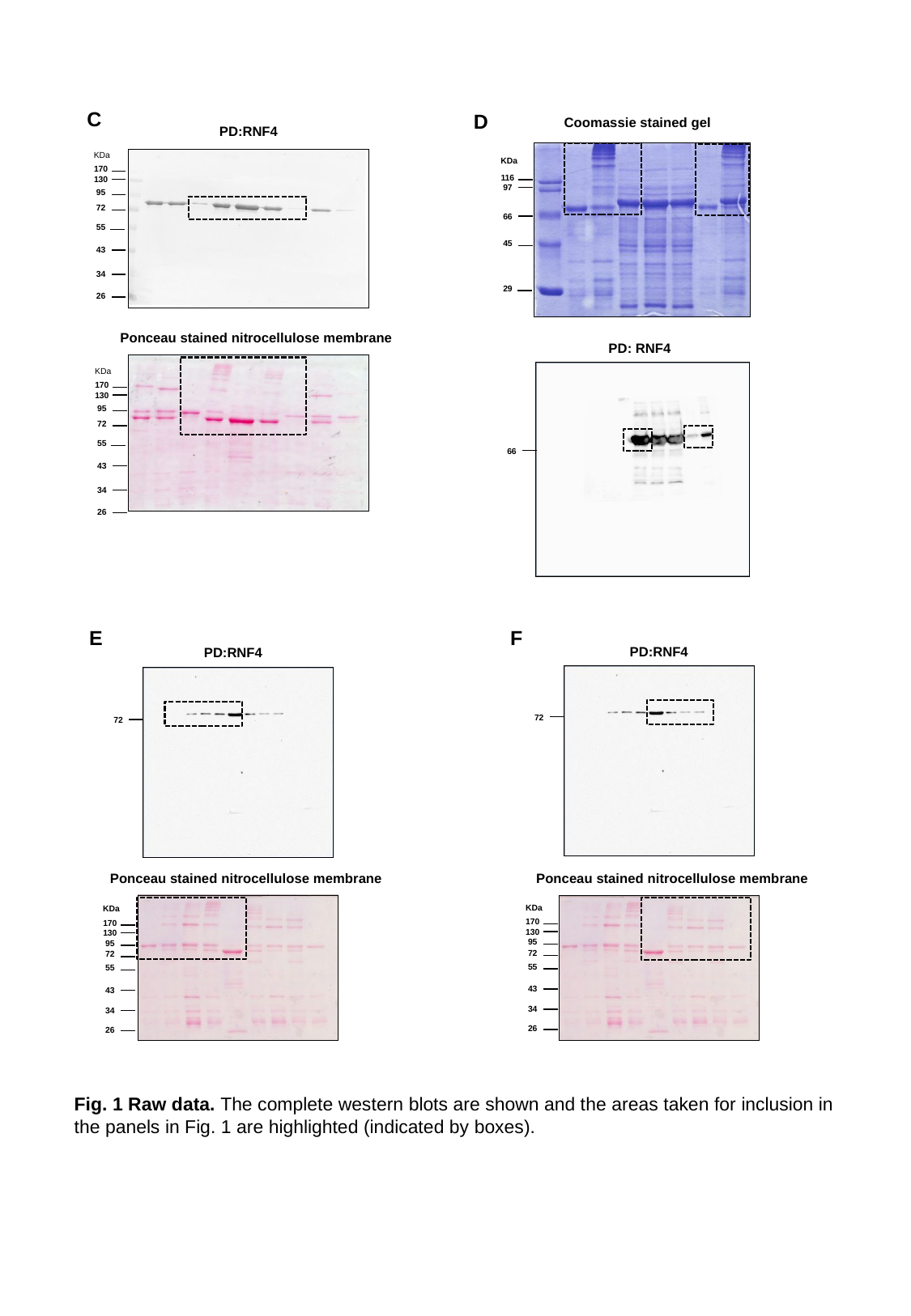

C
D
Coomassie stained gel
PD:RNF4
KDa
KDa
170
116
130
97
95
72
66
55
45
43
34
29
26
Ponceau stained nitrocellulose membrane
PD: RNF4
KDa
170
130
95
72
55
66
43
34
26
E
F
PD:RNF4
PD:RNF4
72
72
Ponceau stained nitrocellulose membrane
Ponceau stained nitrocellulose membrane
KDa
KDa
170
170
130
130
95
95
72
72
55
55
43
43
34
34
26
26
Fig. 1 Raw data. The complete western blots are shown and the areas taken for inclusion in the panels in Fig. 1 are highlighted (indicated by boxes).

## Slide 2
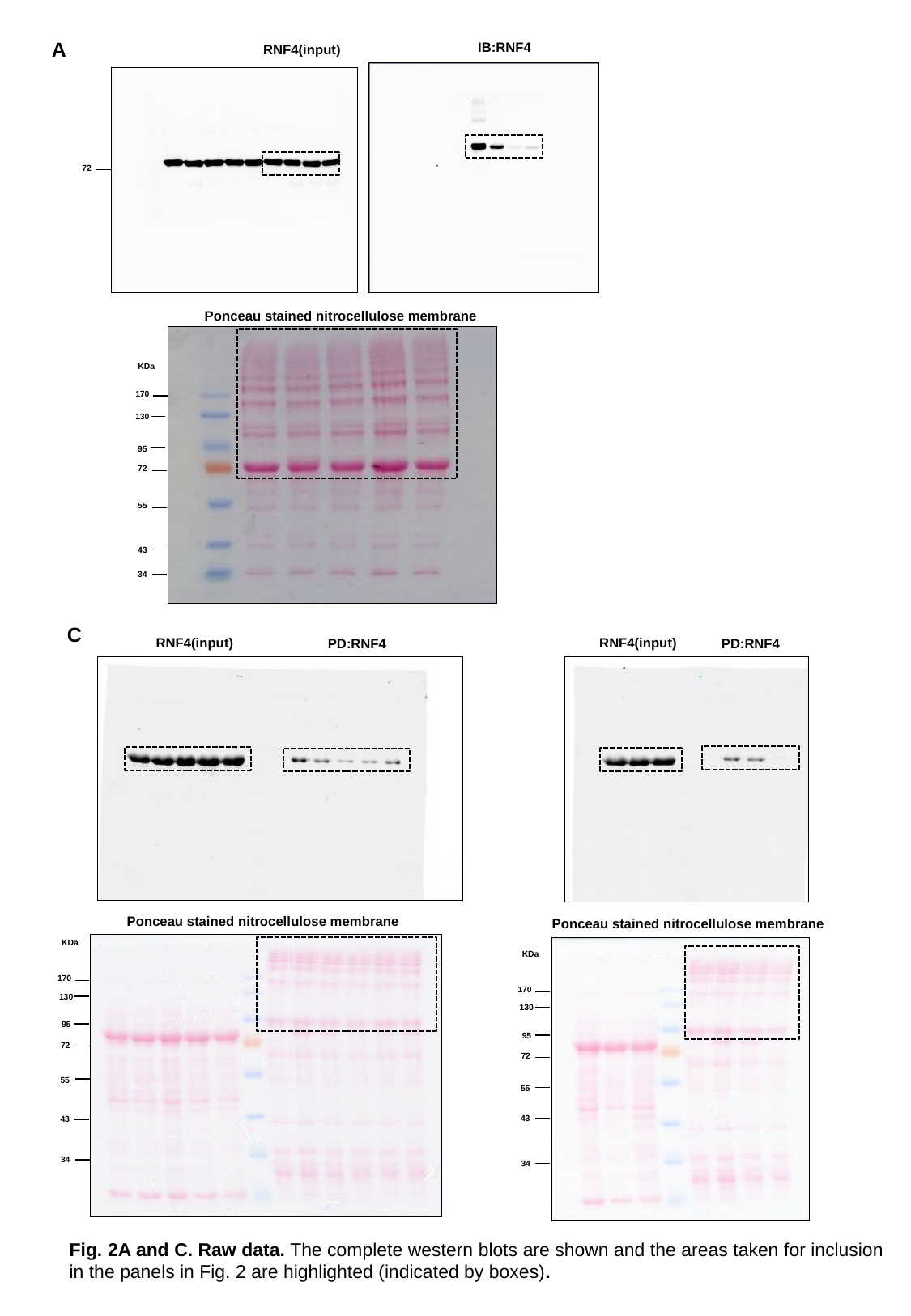

A
IB:RNF4
RNF4(input)
72
Ponceau stained nitrocellulose membrane
KDa
170
130
95
72
55
43
34
C
RNF4(input)
RNF4(input)
PD:RNF4
PD:RNF4
Ponceau stained nitrocellulose membrane
Ponceau stained nitrocellulose membrane
KDa
KDa
170
170
130
130
95
95
72
72
55
55
43
43
34
34
Fig. 2A and C. Raw data. The complete western blots are shown and the areas taken for inclusion
in the panels in Fig. 2 are highlighted (indicated by boxes).

## Slide 3
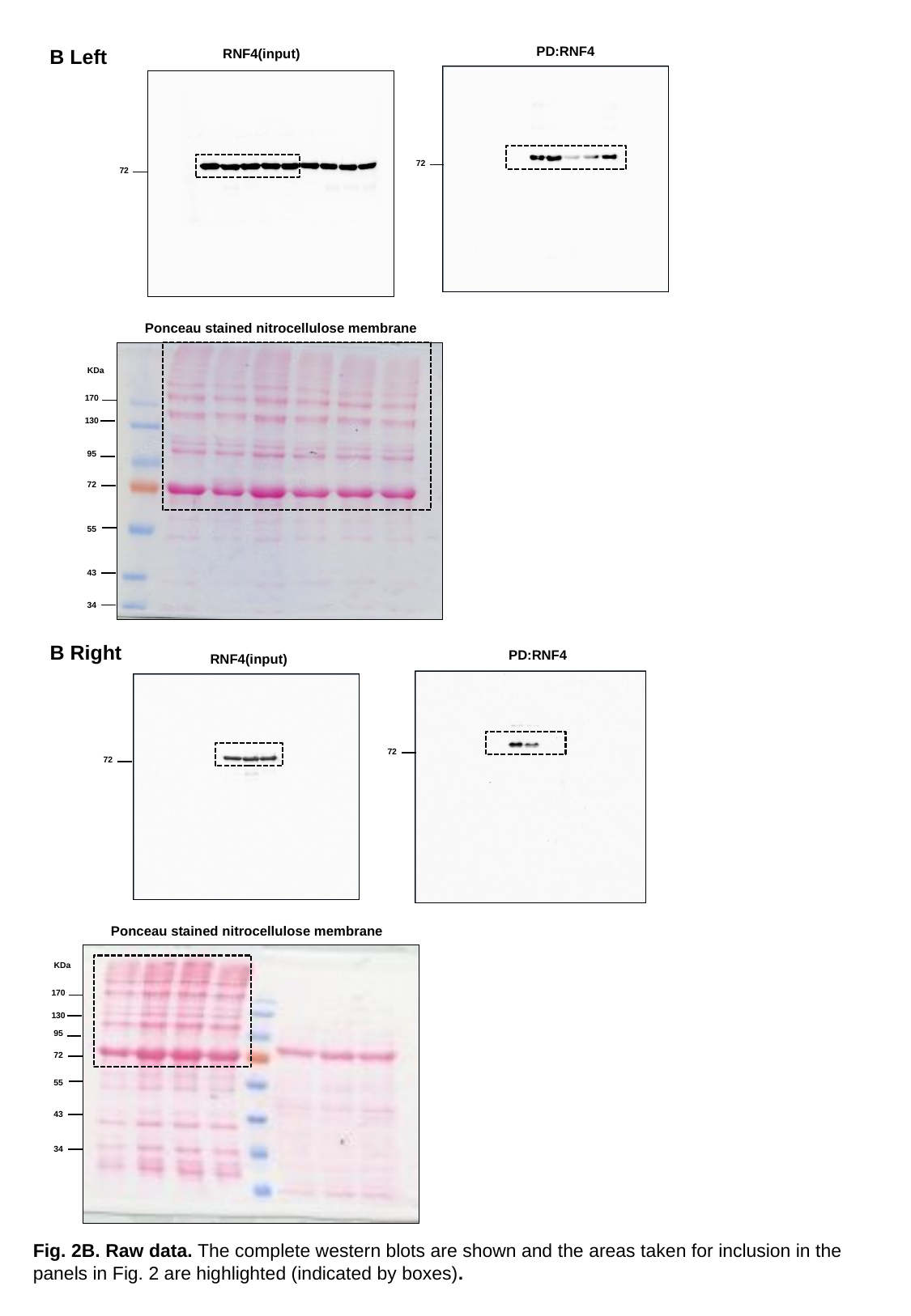

PD:RNF4
B Left
RNF4(input)
72
72
Ponceau stained nitrocellulose membrane
KDa
170
130
95
72
55
43
34
B Right
PD:RNF4
RNF4(input)
72
72
Ponceau stained nitrocellulose membrane
KDa
170
130
95
72
55
43
34
Fig. 2B. Raw data. The complete western blots are shown and the areas taken for inclusion in the panels in Fig. 2 are highlighted (indicated by boxes).
