# Supplementary material for: RNF4 interacts with multiSUMOylated ETV4
Source: Wellcome Open Res. 2017 Feb 17;1:3. Originally published 2016 Nov 15. [Version 2] doi: 10.12688/wellcomeopenres.9935.2 (PMC5445624; doi:10.12688/wellcomeopenres.9935.2)
Supplement: Peer review history

# Peer review: Referee response for version 2

**DOI:** 10.21956/wellcomeopenres.11629.r20342

**Published:** 2017-02-17

**Authors:** Manuel S. Rodriguez

**Affiliations:** 1 Institut des Technologies Avancées en Sciences du Vivant (ITAV), Université de Toulouse, Toulouse, France

Authors have well addressed most comments and suggestions. I am happy to accept this article for indexing.

I have read this submission. I believe that I have an appropriate level of expertise to confirm that it is of an acceptable scientific standard.

# Peer review: Referee response for version 1

**DOI:** 10.21956/wellcomeopenres.10706.r18378

**Published:** 2016-12-09

**Authors:** Manuel S. Rodriguez, Elisa Da Silva-Ferrada

**Affiliations:** 1 Institut des Technologies Avancées en Sciences du Vivant (ITAV), Université de Toulouse, Toulouse, France; 2 Molecular Biology and Biomedicine, University of Coimbra, Coimbra, Portugal

The manuscript RNF4 interacts with multiSUMOylated ETV4 by Aguilar-Martinez *et al* describes that multiSUMOylation of a target protein (ETV4) can be equally well recognised by RNF4, offering an alternative recruitment mechanism to the well known polySUMOylation signal that is recognised by this SUMO-dependent ubiquitin ligase. Authors propose this polySUMO/multiSUMO RNF4 recruitment as a widespread mechanism for this ubiquitin ligase. Further experiments will be required to conclude on the possible molecular and biological differences between the recruitment of RNF4 through poly vs multiple SUMOylation signals.

 The authors present a clear set of data indicating that SUMOylation of ETV4 is required for the interaction of RNF4. In this interaction the SUMO-interacting motifs (SIM) 2 and 3 of RNF4 play a critical role. However, one of the main data supporting the role of the multi-SUMOylation (Figure 1C) lacks quality. In fact, its difficult to observe multi-SUMOylated material in the condition with SUMO3 (K11R). Since the “in bacteria” modification system is not very efficient in terms of production of SUMOylated proteins, perhaps an “in vitro” all recombinant SUMOylation system can be used instead to pull down RNF4 WT. In such experiment ETV4 modified with SUMO3 vs SUMO3(K11R) can be compared. Alternatively, authors can use the same conditions than Figure 1B to pull-down recombinant RNF4. This data will support the actual information.

 Minor comments and suggestions:

- Molecular weight markers should be included in each Western-blot to estimate the size of the poly or mono-SUMOylated forms of ETV4.
- Please justify the use of PMA, including its full name and distributor
- It would be nice to try other STUBL and check if the interaction with multiSUMOylated ETV4 is similar to RNF4.

We have read this submission. We believe that we have an appropriate level of expertise to confirm that it is of an acceptable scientific standard, however we have significant reservations, as outlined above.

# Response

**Published:** 2017-01-26

**Authors:** Andrew D Sharrocks

**Affiliations:** Faculty of Life Sciences, University of Manchester, UK

Our point by point response is indicated below:

 (1) The authors present a clear set of data indicating that SUMOylation of ETV4 is required for the interaction of RNF4. In this interaction the SUMO-interacting motifs (SIM) 2 and 3 of RNF4 play a critical role. However, one of the main data supporting the role of the multi-SUMOylation (Figure 1C) lacks quality. In fact, its difficult to observe multi-SUMOylated material in the condition with SUMO3 (K11R). Since the “in bacteria” modification system is not very efficient in terms of production of SUMOylated proteins, perhaps an “in vitro” all recombinant SUMOylation system can be used instead to pull down RNF4 WT. In such experiment ETV4 modified with SUMO3 vs SUMO3(K11R) can be compared. Alternatively, authors can use the same conditions than Figure 1B to pull-down recombinant RNF4. This data will support the actual information.

**Response**: The referee indicates that the amount of SUMOylation present from the bacterial expression system is low, and therefore provides low stoichiometry which could be improved. However, the critical thing is whether this level of SUMOylation is sufficient to mediate RNF4 binding. We have now included two additional lanes from the original gel that proves that this is the case and we have sufficient SUMOylation. These lanes include a non-SUMOylatable ETV4 derivative that lacks four consensus SUMOylation motifs, and therefore lacks evidence of any multi- and poly-SUMOylation. This mutant ETV4 binds very little RNF4, thereby demonstrating the importance of multi-SUMOylation in this binding event.

 Minor comments and suggestions:

 • Molecular weight markers should be included in each Western-blot to estimate the size of the poly or mono-SUMOylated forms of ETV4.

**Response**: These have now been added throughout the paper and help clarify the nature of the bands we observe.

 • Please justify the use of PMA, including its full name and distributor

**Response**: We have used this as we previously showed that this enhances ETV4 SUMOylation (now indicated in the text). The definition of the acronym has now been added to the materials and methods section.

 • It would be nice to try other STUBL and check if the interaction with multiSUMOylated ETV4 is similar to RNF4.

**Response**: We agree with this and is something that will be of interest for others to pursue in the future.

# Peer review: Referee response for version 1

**DOI:** 10.21956/wellcomeopenres.10706.r17628

**Published:** 2016-11-28

**Authors:** Tony Hunter

**Affiliations:** 1 Molecular and Cell Biology Laboratory, Salk Institute for Biological Studies, La Jolla, CA, USA

Here the authors have extended their studies of sumoylation of the ETS family transcription factor, ETV4. They have previously reported that ETV4 K96, K222 and K256, which lie in sumoylation consensus sequences, are sumoylated, and shown that sumoylated ETV4 is a target for the RNF4 SUMO-targeted E3 ubiquitin ligase (STUbL). Here, they demonstrated that ETV4 is multi-sumoylated when co-expressed in HEK293T cells together with UBE2/UBC9, PIAS4 and WT SUMO3 or K11R SUMO3. Since K11R SUMO3 cannot form SUMO chains, they deduced that the slowly migrating sumoylated forms of ETV4 detected contain mono-SUMO3 adducts at multiple sites rather than SUMO chains. Bead-bound sumoylated GST-ETV4 brought down RNF4 efficiently, whereas mutant GST-ETV4 lacking pairs of the identified sumoylation sites bound less RNF4, and mutant GST-ETV4 lacking 4 SUMO sites (including K328) brought down no detectable RNF4. Next they assessed the importance of the individual SIMs in the RNF4 tetra-SIM array for binding multi-sumoylated ETV4, by mutating SIM consensuses singly and in combination, concluding that the central SIM2 and SIM3 motifs contribute most significantly to the binding of RNF4 to multi-sumoylated ETV4, as others have found for RNF4 binding to poly-SUMO chains. They conclude that RNF4 can recognize multi-monosumoylated proteins through its SIM array, in addition to proteins with poly-SUMO chains.

 Overall, the experiments are described in sufficient detail and the data are of good quality, providing strong experimental support for their conclusion that RNF4 can bind to multi-monosumoylated proteins through its array of four SIMs. This theoretical possibility had been proposed by others (e.g. Sun and Hunter (2012)), but it is important to have direct experimental validation of this concept, since the ability of RNF4 to bind to multi-monosumoylated proteins expands the range of possible targets for RNF4 STUbL-mediated ubiquitylation. One point that needs further discussion is the likely topology of binding of the RNF4 SIMs to a multi-monosumoylated protein such as ETV4. For instance, the schematic in panel D is not accurate in terms of the relative size of the sumoylated region in ETV4 and the size of the SIM domain in RNF4. Presumably, this region of ETV4 is flexible, but there would still need to be looping out of sequences between two adjacent SUMO residues for them to be able to bind simultaneously to SIMs 2 and 3 in a single RNF4 molecule. Some discussion of this point is merited.

 Other points:

1. Figure 1: The overall stoichiometry of ETV4 sumoylation in transfected HEK293 cells shown in panel B was low even under conditions designed to maximize sumoylation, i.e. there was a lot of unmodified ETV4. This might suggest that ETV4 sumoylation is cooperative, in the sense that sumoylated ETV4 molecules may be more susceptible than unmodified ETV4 to further sumoylation at additional sites. The patterns of sumoylated ETV4 bands in panels C and D which were obtained by co-expression of GST-ETV4 and the sumoylation machinery in bacteria are quite different from the pattern in panel B obtained in mammalian cells, and given the apparent size of the shift  of the bands near the top of the gel in the WT SUMO3 sample it seems likely that SUMO3 chains were attached, as the authors suggest. Unfortunately, the Ponceau stain is rather faint and it is hard to tell what bands are present in the K11R SUMO3 sample. Although the data in panel D suggest that ETV4 sumoylation site specificity is similar in bacterial and mammalian cells, one would like to see bacterial samples run side by side with sumoylated GST-ETV4 from mammalian cells (is it clear that there are no SUMO sites in GST?), and some discussion of sumoylation specificity in this bacterial system is desirable (the stoichiometry seems significantly higher than in mammalian cells), and in some ways it is surprising that adventitious sumoylation did not occur at nonconsensus sites in ETV4, particularly in the absence of SUMO proteases in the system. Did the authors confirm which Lys were sumoylated in the bacterial system using mass spec? Ideally, the experiments in panels D and E should be repeated with K11R SUMO3 to formally establish that RNF4 binding is not dependent to any extent on SUMO chain formation.
2. Figure 2: The patterns of sumoylated ETV4 look different from those in Figure 1C-E. The patterns with WT SUMO3 could be due to polysumoylation, but the bands seem to be rather discrete for this, and there are more than 4 bands even with K11R SUMO3, as seen in panel C. Admittedly this could be because a single SUMO at different sites in ETV4 results in a different gel mobility, but this issue ought to be discussed.
3. With regard to the topology of the binding of the RNF4 SIM array to SUMO chains or to multiple single SUMOs, further discussion of the structural insights into RNF4 SIM/SUMO interactions reported in Xu et al. (2014), and the importance of SIMs 2 and 3 in this interaction, is warranted.

 

 Minor points:

1. In Figure 1A RNF4 is depicted with only 3 SIMs, possibly because ETV4 is shown with three SUMO sites or a SUMO chain with three residues. In contrast, RNF4 in Figure 2D is depicted with 4 SIMs in Figure 2D, which is the correct number of SIMs. I recommend changing Figure 1A to be consistent with Figure 2D. In addition, the legend to Figure 1 should indicate very clearly that the samples in panels C-E were obtained with ETV4 sumoylated in bacteria.
2. In the experiments in Figure 2, MBP-RNF4 was used. Was MBP-RNF4 also used in the experiments in Figure 1C-E. If so this should be indicated? If not, how was recombinant RNF4 purified for this experiment - only MBP-RNF4 is described in the Methods.

I have read this submission. I believe that I have an appropriate level of expertise to confirm that it is of an acceptable scientific standard.

# Response

**Published:** 2017-01-26

**Authors:** Andrew D Sharrocks

**Affiliations:** Faculty of Life Sciences, University of Manchester, UK

Our point by point response to the issues raised is provided below:

 One point that needs further discussion is the likely topology of binding of the RNF4 SIMs to a multi-monosumoylated protein such as ETV4. For instance, the schematic in panel D is not accurate in terms of the relative size of the sumoylated region in ETV4 and the size of the SIM domain in RNF4. Presumably, this region of ETV4 is flexible, but there would still need to be looping out of sequences between two adjacent SUMO residues for them to be able to bind simultaneously to SIMs 2 and 3 in a single RNF4 molecule. Some discussion of this point is merited.

**Response**: This is an important point and we have now added brief discussion of this in the manuscript.

 Other points:

 1. Figure 1: The overall stoichiometry of ETV4 sumoylation in transfected HEK293 cells shown in panel B was low even under conditions designed to maximize sumoylation, i.e. there was a lot of unmodified ETV4. This might suggest that ETV4 sumoylation is cooperative, in the sense that sumoylated ETV4 molecules may be more susceptible than unmodified ETV4 to further sumoylation at additional sites. The patterns of sumoylated ETV4 bands in panels C and D which were obtained by co-expression of GST-ETV4 and the sumoylation machinery in bacteria are quite different from the pattern in panel B obtained in mammalian cells, and given the apparent size of the shift  of the bands near the top of the gel in the WT SUMO3 sample it seems likely that SUMO3 chains were attached, as the authors suggest. Unfortunately, the Ponceau stain is rather faint and it is hard to tell what bands are present in the K11R SUMO3 sample. Although the data in panel D suggest that ETV4 sumoylation site specificity is similar in bacterial and mammalian cells, one would like to see bacterial samples run side by side with sumoylated GST-ETV4 from mammalian cells (is it clear that there are no SUMO sites in GST?), and some discussion of sumoylation specificity in this bacterial system is desirable (the stoichiometry seems significantly higher than in mammalian cells), and in some ways it is surprising that adventitious sumoylation did not occur at nonconsensus sites in ETV4, particularly in the absence of SUMO proteases in the system. Did the authors confirm which Lys were sumoylated in the bacterial system using mass spec? Ideally, the experiments in panels D and E should be repeated with K11R SUMO3 to formally establish that RNF4 binding is not dependent to any extent on SUMO chain formation.

**Response**: We added an additional panel to Fig.1 to compare binding to WT and K11R versions of SUMO. This confirms that SUMO chain formation is not needed for RNF4 binding (new Fig. 1F). We have not performed mass spectrometry on the bacterially generated SUMOylated ETV4. However, Fig. 1E and F demonstrate that removal of the mapped SUMOylation sites in ETV4 eliminates ETV4 SUMOylation in the bacterial expression system and this loss of SUMOylation results in the loss of the associated RNF4 binding ability. We altered the text of the paper to emphasise this point.

 2. Figure 2: The patterns of sumoylated ETV4 look different from those in Figure 1C-E. The patterns with WT SUMO3 could be due to polysumoylation, but the bands seem to be rather discrete for this, and there are more than 4 bands even with K11R SUMO3, as seen in panel C. Admittedly this could be because a single SUMO at different sites in ETV4 results in a different gel mobility, but this issue ought to be discussed.

**Response**: The differences in mobility likely result from differences in the mobility of branched chain SUMO conjugates, as the size of the ETV4 construct varies. Fig. 1B contains Flag staged ETV4 but Fig. 1D contains GST-ETV4(1-335) and Fig. 1C, E and F contain GST-ETV4(full-length). As the referee points out, the different bands can be the result of multiple different combinations of SUMO modifications where either single or multiple SUMO molecules are attached. We have now discussed this more in the paper.

 3. With regard to the topology of the binding of the RNF4 SIM array to SUMO chains or to multiple single SUMOs, further discussion of the structural insights into RNF4 SIM/SUMO interactions reported in Xu et al. (2014), and the importance of SIMs 2 and 3 in this interaction, is warranted.

**Response**: We have now added further discussion of this and how the structure reveals a more substantive role for SIM3 than previously appreciated.

 Minor points:

 1. In Figure 1A RNF4 is depicted with only 3 SIMs, possibly because ETV4 is shown with three SUMO sites or a SUMO chain with three residues. In contrast, RNF4 in Figure 2D is depicted with 4 SIMs in Figure 2D, which is the correct number of SIMs. I recommend changing Figure 1A to be consistent with Figure 2D. In addition, the legend to Figure 1 should indicate very clearly that the samples in panels C-E were obtained with ETV4 sumoylated in bacteria.

**Response**: We have now harmonised the two schematics to more accurately depict the interactions and protein structures under investigation. The legend to Fig. 1 has been modified to indicate the source of theSUMOylated GST-ETV4 proteins.

 2. In the experiments in Figure 2, MBP-RNF4 was used. Was MBP-RNF4 also used in the experiments in Figure 1C-E. If so this should be indicated? If not, how was recombinant RNF4 purified for this experiment - only MBP-RNF4 is described in the Methods.

**Response**: MBP-RNF4 was used throughout and this is now clearly indicated in the legend to Fig.1.
